# Design and validation of the Health Professionals' Attitudes Toward the Homeless Inventory (HPATHI)

**DOI:** 10.1186/1472-6920-5-2

**Published:** 2005-01-10

**Authors:** David S Buck, F Marconi Monteiro, Suzanne Kneuper, Donna Rochon, Dana L Clark, Allegra Melillo, Robert J Volk

**Affiliations:** 1 Department of Family and Community Medicine, Baylor College of Medicine, Houston, TX, USA; 2 Department of Family Practice, University of California San Francisco, San Francisco, CA, USA

## Abstract

**Background:**

Recent literature has called for humanistic care of patients and for medical schools to begin incorporating humanism into medical education. To assess the attitudes of health-care professionals toward homeless patients and to demonstrate how those attitudes might impact optimal care, we developed and validated a new survey instrument, the Health Professional Attitudes Toward the Homeless Inventory (HPATHI). An instrument that measures providers' attitudes toward the homeless could offer meaningful information for the design and implementation of educational activities that foster more compassionate homeless health care. Our intention was to describe the process of designing and validating the new instrument and to discuss the usefulness of the instrument for assessing the impact of educational experiences that involve working directly with the homeless on the attitudes, interest, and confidence of medical students and other health-care professionals.

**Methods:**

The study consisted of three phases: identifying items for the instrument; pilot testing the initial instrument with a group of 72 third-year medical students; and modifying and administering the instrument in its revised form to 160 health-care professionals and third-year medical students. The instrument was analyzed for reliability and validity throughout the process.

**Results:**

A 19-item version of the HPATHI had good internal consistency with a Cronbach's alpha of 0.88 and a test-retest reliability coefficient of 0.69. The HPATHI showed good concurrent validity, and respondents with more than one year of experience with homeless patients scored significantly higher than did those with less experience. Factor analysis yielded three subscales: Personal Advocacy, Social Advocacy, and Cynicism.

**Conclusions:**

The HPATHI demonstrated strong reliability for the total scale and satisfactory test-retest reliability. Extreme group comparisons suggested that experience with the homeless rather than medical training itself could affect health-care professionals' attitudes toward the homeless. This could have implications for the evaluation of medical school curricula.

## Background

In 2003, the Department of Health and Human Services reported that there are between two to three million people in the United States who experience homelessness each year [1]. According to Gelberg and Arangua [2], such estimates provide only a partial picture of the problem: among the U.S. population, 14% (26 million people) have been homeless at some time in their lives and 5% (8.5 million people) have been homeless within the past five years. Yet even as this number grows, the homeless continue to be subjected to broad stereotyping and stigmatization, both of which make it easier to ignore them. It is not surprising then that homeless persons are reluctant to obtain needed, continuous medical care within traditional outpatient settings. This is particularly problematic because they often have competing or immediate needs [3,4], multiple health problems [5,6], and increased morbidity and mortality [7,8].

The disinclination of the homeless to seek care may be due in part to the way in which many health-care workers respond to them. A less investigated but possibly equally important circumstance is the attitudes that health-care professionals have toward the homeless. As members of the larger society, these professionals often harbor the same preconceived ideas and biases toward the homeless that the rest of society does. Such judgmental attitudes can, and often do, emerge during the provider-patient encounter, thus limiting the effectiveness of medical treatment of the homeless [9].

As a countermeasure to prevailing negative attitudes and widespread stigmatization toward marginalized persons, recent medical literature has drawn on a well-known practice in the social sciences [10,11] and has begun calling for greater emphasis on the dimensions of compassion and humanism in medical education [12-14]. Humanism in psychology became popular in the 1950s when Rogers [15] began practicing client-centered therapy, which allows the relationship between therapist and client to develop so that the client can be guided within the framework of the therapeutic encounter. According to Branch [[16], p. 1067], humanism in medicine may be defined as "the physicians' attitudes and actions that demonstrate interest in and respect for the patient, and address the patient's concerns and values." As physicians interact with homeless patients, a heightened sense of these patients' vulnerability may evoke greater empathy and humanism, two attitudes that can only have a positive impact on the quality of care that the physicians provide.

Although it would be helpful to have a better understanding of physicians' current opinions about homeless patients, only two instruments have been designed and validated to measure attitudes toward homeless persons: the Attitudes Toward the Homeless Inventory (ATHI) [17], and the Attitudes Towards the Homeless Questionnaire (ATHQ) [18]. The ATHI surveys American college students' attitudes toward homelessness but does not specifically address the attitudes of health-care providers. In addition, it was not designed to measure a health-care provider's desire or confidence in his or her ability to deliver health care to homeless persons. Nevertheless, Buchanan et al. [19] recently used the ATHI to measure the attitudes of 12 primary care residents before and after a 2-week rotation in homeless health care and found that they felt more comfortable affiliating with homeless people after the course. The ATHQ, which was designed and validated in the United Kingdom, does assess the attitudes of health-care providers toward the homeless, but differences in health systems terminology (e.g., NHS for National Health Service, and terms related to homelessness, such as sleep rough) would make its use in its original form problematic in the United States. For this reason, we decided to use the ATHI for comparison purposes because its language is more consistent with American English.

The purpose of our study was to develop and validate a new measure, the Health Professionals' Attitude Toward the Homeless Inventory (HPATHI), an instrument that could be used to assess United States medical students' and physicians' attitudes toward homeless persons and to measure their level of interest and confidence in their ability to deliver health-care services to the homeless population. The objectives of this study were to: 1) describe the process of designing and validating the new instrument, and 2) discuss the usefulness of the instrument for assessing the impact of educational experiences working with the homeless on the attitudes, interest, and confidence of medical students and other health-care professionals.

## Methods

The development and validation of the HPATHI occurred in three phases. The first phase consisted of the identification of items for the inventory; the second phase involved a pilot test of the initial instrument with a group of medical students; and the final phase entailed modifying the instrument and administering it in its revised form to our target population – health-care professionals, including primary care physicians, primary care residents, and third-year medical students.

Throughout this three-phase process, we sought to ascertain the reliability and validity of the HPATHI. Three types of validity were tested. Content validity was evaluated using the Delphi technique [20], which seeks consensus on instrument items among a panel of experts. Concurrent validity was computed by correlating the target population's responses to the HPATHI with their responses to the ATHI. Construct validity was established by using the extreme groups method [21], by conducting an exploratory factor analysis, and by conducting item analyses to assess the relationship of each individual item to the instrument's overall scale and hypothetical subscales.

### Stages of development and validation of the HPATHI

In Phase 1, a group of physicians and nurse practitioners, all experts in homeless health care, was recruited through snowball sampling [22] to serve on a Delphi panel [20]. These individuals were identified as experts because they work with the homeless on a regular basis and are members of the National Health Care for the Homeless Clinicians' Network. They received a list of statements about the homeless, homelessness, and homeless health care that was compiled by selecting and adapting appropriate items from the AHTI [17] and the ATHQ [18]. We, as the authors of this study, added additional items that were based on our experiences and our review of the literature. The experts were asked to classify the statements into one of three categories ("essential," "interesting but not essential," and "irrelevant") and to generate other items that they considered to be necessary to explore health-care professionals' attitudes toward the homeless. The panel reached a consensus on the items to be used in the instrument through successive rankings of the items. The statements were then combined into an instrument with a five-point Likert scale (1 = strongly disagree; 2 = disagree; 3 = neither agree nor disagree; 4 = agree; and 5 = strongly agree) organized around three thematic areas: a) *attitudes *toward the homeless; b) *interest *in working with the homeless; and c) *confidence *in one's ability to work with the homeless.

During Phase 2, we administered the first draft of the HPATHI to a convenience sample of third-year medical students enrolled at Baylor College of Medicine (BCM) in Houston, Texas. These students were selected because they showed an interest in working with the homeless by registering for an elective course that allows students to provide health care to the homeless in a clinic setting [23]. Of the 100 students who attended class on the day that the instrument was administered, 72 completed it and were asked to provide contact information for the retest. Student responses to the instrument were analyzed using Cronbach's alpha coefficient to establish its internal consistency. Two weeks later, 34 of the 63 students who provided e-mail addresses completed the instrument a second time, thereby providing the data to determine the HPATHI's test-retest reliability. We then conducted an item analysis of redundant items or those with poor item-to-scale correlations.

In Phase 3, we prepared Web-based versions of the HPATHI and of the ATHI and sent the Internet link to our target population with a standard invitation to complete both instruments. Primary care physicians who serve as faculty in the BCM Department of Family and Community Medicine, family practice residents in the BCM Department of Family and Community Medicine, general internal medicine residents in the BCM Department of Family and Community Medicine, and students from the BCM Medical School served as a convenience sample of individuals completing the instrument over a six-month period. Subsequent data analysis consisted of:

1. Conducting an exploratory factor analysis using a Promax rotation to examine the structure of the HPATHI.

2. Estimating the internal consistency reliability of the online version of the HPATHI using Cronbach's alpha coefficient;

3. Determining the concurrent validity of the HPATHI against the ATHI;

4. Comparing extreme groups on their responses to the HPATHI. The extreme groups were determined according to their level of training (preclinical medical students vs. physicians) and according to their experience in working with the homeless (<1 month vs. >1 year).

This study was approved by the BCM Institutional Review Board for educational research with human subjects. Data analysis was conducted using SPSS Version 12.0 statistical software for Phases 1 and 2 and SAS^® ^(V8.2 and V9.1) for Phase 3.

## Results

### Phase 1: instrument development

Of the 23 panel members who received the first list of 24 statements, 16 (70%) reviewed them, rank ordered them, and generated an additional 26 statements. We rearranged the combined list of 50 statements according to the proposed ranking ("essential," "interesting but not essential," and "irrelevant") and returned them to the 16 panel members for a second ranking. Based on their responses, six statements were eliminated and the remaining 44 were returned to the 16 panel members for a third ranking. Nine panel members returned responses after this iteration, and we eliminated nine additional items according to their recommendations. The remaining 35 items then constituted the first draft of the instrument we named the Healthcare Professionals' Attitude Toward the Homeless Inventory (HPATHI). Although seven panel members did not participate in the third iteration, their rankings in the second iteration corresponded to the final list of items.

### Phase 2: pilot administration of the 35-item HPATHI

The sample population of third-year medical students completed a pilot administration of the 35-item HPATHI; the subset of students who responded two weeks later underwent a second administration of the same instrument. Table 1 displays the means and standard deviations of student responses to both administrations of the HPATHI. Items 2, 5, 6, 11, 15, 16, 20, and 23 were reverse-coded for the analysis so that a higher total mean on the instrument would indicate a positive attitude toward the homeless.

**Table 1:** Means and standard deviations for HPATHI* test and retest

***Statement***	***Test n = 76***		***Retest n = 34***	
	
	***M***	***SD***	***M***	***SD***
1. Homeless people are victims of circumstance.	3.26	.839	3.24	.819
2. Most homeless people are mentally ill.	2.90	.875	3.24	.955
3. Homeless people have the right to basic health care.	4.60	.620	4.68	.475
4. Homelessness is a major problem in our society.	4.36	.698	4.50	.707
5. Homeless people choose to be homeless.	3.71	.777	3.91	.712
6. Homeless people are lazy.	3.82	.738	3.94	.694
7. Health care dollars should be directed toward serving the poor and homeless.	3.83	.888	4.03	.717
8. Doctors should address the physical and social problems of the homeless.	4.17	.839	4.47	.563
9. Doctors have a duty to care for the homeless.	3.94	.977	4.18	.834
10. Caring for the homeless is pointless since they do not follow-up.	4.14	.612	4.24	.654
11. Providing medical care for the homeless is futile.	4.04	.740	4.21	.592
12. I am comfortable being a primary care provider for a homeless person with a major mental illness.	3.03	1.14	3.44	1.05
13. I feel comfortable being part of a team when providing care to the homeless.	4.32	.535	4.15	.643
14. I feel comfortable providing care to different minority and cultural groups.	4.42	.710	4.18	.673
15. I feel overwhelmed by the complexity of the problems that homeless people have.	2.97	.839	3.09	.996
16. I understand that my patients' priorities may be more important than following my medical recommendations.	3.97	.769	4.24	.741
17. I entered medicine because I want to help those in need.	4.42	.687	4.62	.551
18. I am interested in working with the underserved.	3.96	.941	3.88	.946
19. I enjoy addressing psychosocial issues with patients.	3.69	.973	3.74	1.08
20. I resent the amount of time it takes to see homeless patients.	3.79	.604	3.94	.547
21. I enjoy learning about the lives of my homeless patients.	3.58	.921	3.82	.834
22. I believe social justice is an important part of health care.	3.75	1.06	3.74	1.14
23. I believe caring for the homeless is not financially viable for my career.	3.24	.831	3.26	.864
24. I am too pressed for time to investigate psychosocial issues routinely.	3.37	.941	3.56	.894
25. I feel overwhelmed by the number of problems that homeless people have.	2.79	.844	2.88	.844
26. My knowledge regarding the problem of homelessness is adequate.	2.58	.931	2.65	.884
27. I can provide care for the homeless effectively.	2.97	.878	3.00	.921
28. Homeless people come from all walks of life.	4.42	.622	4.65	.485
29. Most homeless people tend to be drug addicts or alcoholics.	3.10	.735	3.38	.697
30. I think mentally ill homeless people refuse to get treatment.	3.67	.692	3.82	.797
31. Homeless people are dangerous, aggressive, and physically threatening.	3.97	.556	3.97	.627
32. There are only a few children among the homeless population.	4.31	.493	4.32	.684
33. All people have a right to basic health care.	4.50	.805	4.59	.657
34. I feel it is important to provide care to all socio-economic groups.	4.54	.670	4.62	.604
35. Most poor people have adequate access to health care through the public system.	2.15	1.02	2.03	.937
***Totals***	114	6.93	116	7.75

* Statements 24–35 were excluded from HPATHI after analysis.

Cronbach's alpha coefficient for the first administration was 0.87; the test-retest reliability coefficient (Pearson r) was 0.69. Through an item analysis we discarded 12 items that were either highly correlated with other items, and were thus considered repetitious, or that had item-scale correlations less than 0.20.

### Phase 3: administration of the HPATHI to the target population

One hundred and sixty health-care professionals (24 primary care physicians, 15 primary care residents, 47 clinical medical students, 71 preclinical medical students, and 3 medical students who did not specify their education level) from one academic institution completed the HPATHI; 147 of them also completed the ATHI. Table 2 displays the means and standard deviations for both instruments by gender, by level of training (primary care physician; primary care resident; clinical medical student; preclinical medical student), and by experience with the homeless (no experience; <1 month, >1 month but <1 year, and >1 year).

**Table 2:** Comparison of the HPATHI and ATHI by gender, level of training, and experience

	HPATHI			ATHI		
	N	Mean	SD	N	Mean	SD

**Total**	160	3.90	0.34	147	3.36	0.28
						
**Gender**						
Female	103	3.95	0.30	96	3.38	0.26
Male	57	3.81	0.38	51	3.32	0.30
						
**Level of Training†**						
MS1	55	3.89	0.32	54	3.34	0.26
MS2	16	4.10	0.23	16	3.47	0.21
MS3	28	3.91	0.41	26	3.38	0.36
MS4	19	3.84	0.30	18	3.33	0.25
Resident	15	3.73	0.22	13	3.30	0.25
Faculty	24	3.93	0.36	18	3.38	0.30
						
**Experience**						
None	31	3.72	0.33	28	3.21	0.23
<1 month	64	3.91	0.30	62	3.37	0.27
>1 month & <1 year	30	3.88	0.32	25	3.37	0.22
1 – 3 years	17	4.10	0.28	16	3.49	0.31
>3 years	18	4.05	0.38	16	3.46	0.33

^†^On the HPATHI, 3 respondents failed to indicate a level of training and on the ATHI, 2 respondents failed to indicate a level of training.

The exploratory factor analysis (principal components), using a Promax rotation to account for the relationship among the factors, yielded a three-factor structure that explained 39% of the variance of the data. Factor 1 consisted of items 12, 13, 14, 17, 18, 19, 20, 21, 22, and 23 and was labeled Personal Advocacy; factor 2 consisted of items 1, 3, 4, 7, 8, 9, and 15 and was labeled Social Advocacy; and factor 3 consisted of items 5, 6, 10, and 11 and was labeled Cynicism. Table 3 presents the items with their loadings in each factor.

**Table 3:** Factor loadings for the 23-item HPATHI

	**Factor 1**	**Factor 2**	**Factor 3**
1. Homeless people are victims of circumstance.	-0.095	0.518	0.064
3. Homeless people have the right to basic health care.	-0.096	0.644	0.154
4. Homelessness is a major problem in our society.	-0.124	0.677	0.140
5. Homeless people choose to be homeless.	0.008	0.233	0.469
6. Homeless people are lazy.	-0.026	0.330	0.559
7. Health-care dollars should be directed toward serving the poor and homeless.	0.180	0.575	0.079
8. I am comfortable being a primary care provider for a homeless person with a major mental illness.	0.329	0.466	-0.071
9. I feel comfortable being part of a team when providing care to the homeless.	-0.015	0.514	0.188
10. I feel comfortable providing care to different minority and cultural groups.	-0.007	0.152	0.725
11. I feel overwhelmed by the complexity of the problems that homeless people have.	0.028	0.056	0.748
12. I understand that my patients' priorities may be more important than following my medical recommendations.	0.469	-0.206	0.202
13. Doctors should address the physical and social problems of the homeless.	0.438	-0.046	0.395
17. I entered medicine because I want to help those in need.	0.485	0.003	0.088
18. I am interested in working with the underserved.	0.516	0.168	0.085
19. I enjoy addressing psychosocial issues with patients.	0.697	0.106	-0.224
20. I resent the amount of time it takes to see homeless patients.	0.613	-0.214	0.097
21. I enjoy learning about the lives of my homeless patients.	0.788	-0.039	-0.188
22. I believe social justice is an important part of health care.	0.509	0.404	-0.154
23. I believe caring for the homeless is not financially viable for my career.	0.504	-0.096	0.042

The HPATHI was further shortened to 19 items by the deletion of four more items, which either were not represented in the three-factor structure (items 2, 14, and 16) or had an adverse effect on the subscale's reliability (item 15). The three subscales were also significantly related to each other: factor 1 had Pearson's r correlations of 0.47 (n = 160; p < 0.001) with factor 2 and 0.43 (n = 160; p < 0.001) with factor 3; and factor 2 had a Pearson's r correlation of 0.48 (n = 160; p < 0.001) with factor 3. Table 4 displays the descriptive statistics and measurement properties for the 19-item HPATHI total and subscales. These three factors, if taken as subscales for the HPATHI, showed satisfactory Cronbach's alpha coefficients: 0.75, 0.72, 0.72, and 0.84 respectively for factor 1 (mean = 3.86; sd = 0.47), factor 2 (mean = 4.06; sd = 0.46), factor 3 (mean = 4.06; sd = 0.50), and total scale (mean = 3.96; sd = 0.38).

**Table 4:** Measurement properties for reduced HPATHI scale and subscales

Descriptive Statistics				Subscale Statistics		Full-scale Statistics	
Item	Mean	SD	Scale^¶^	Item-Total Correlation	Cronbach's Alpha if Deleted	Item-Total Correlation	Cronbach's Alpha if Deleted

1	2.55	0.76	SA	0.28	0.73	0.27	0.84
3	1.45	0.61	SA	0.52	0.66	0.40	0.83
4	1.68	0.71	SA	0.48	0.67	0.39	0.83
5	2.20	0.80	C	0.42	0.73	0.40	0.83
6	2.14	0.65	C	0.53	0.65	0.50	0.83
7	2.15	0.79	SA	0.48	0.67	0.55	0.82
8	1.81	0.67	SA	0.49	0.67	0.50	0.82
9	2.01	0.72	SA	0.47	0.67	0.39	0.83
10	1.76	0.60	C	0.59	0.62	0.47	0.83
11	1.68	0.64	C	0.54	0.65	0.44	0.83
12	2.82	0.96	PA	0.31	0.75	0.31	0.84
13	1.79	0.58	PA	0.43	0.73	0.47	0.83
17	1.63	0.58	PA	0.38	0.73	0.37	0.83
18	1.84	0.78	PA	0.51	0.71	0.52	0.82
19	2.23	0.97	PA	0.51	0.71	0.47	0.83
20	2.09	0.74	PA	0.40	0.73	0.36	0.83
21	2.19	0.78	PA	0.55	0.71	0.46	0.83
22	2.00	0.85	PA	0.49	0.71	0.54	0.82
23	2.66	0.94	PA	0.34	0.74	0.31	0.84

^¶^PA = Personal Advocacy; SA = Social Advocacy; C = Cynicism

The Pearson's correlation coefficient between the HPATHI and the ATHI was 0.68 for the HPATHI's total scale (concurrent validity) (Table 4). For the extreme group comparisons, no significant difference was found between preclinical medical students and primary care physicians in their responses to the HPATHI (F = 1.05; df = 3, 156; p = 0.371). On the other hand, respondents who had more than one year of experience with the homeless scored significantly higher than those who had less than one month of experience (F = 6.19; df = 2, 157; p = 0.003) (Table 5). When the individual hypothetical subscales were considered, all items were either moderately or strongly correlated with their respective subscales (range of Pearson's correlation coefficients, 0.38 to 0.68). However, when the entire instrument was considered, the item analysis showed that items 1, 2, 15, and 16 had low item-scale correlations (Pearson's r < 0.24).

**Table 5:** Extreme group comparisons by level of training and experience with the homeless

	**N**	**Mean**	**SD**
**Level of Training**			
MS1 & MS2	71	3.94	0.31
Residents & Faculty	39	3.85	0.33
			
**Experience with the Homeless‡**			
Less than 1 month	95	3.85	0.32
More than 1 year	35	4.01	0.33

^‡^Differences between the means of the two groups by experience were statistically significant (p < 0.01)

## Discussion

Using the Delphi method to select the survey items helped ensure the HPATHI's content validity. Not only were the items selected and validated by homeless health-care experts, but the items chosen for inclusion in the HPATHI were based on findings from current literature on the issue. In addition, by correlating the HPATHI with the ATHI, we were able to demonstrate satisfactory concurrent validity. Construct validity for the HPATHI (i.e., attitudes toward the homeless) was determined by the extreme group comparisons, the item analyses, and the factor analysis.

Results of the extreme group comparisons showed that experience with the homeless, rather than medical training, is a significant factor that correlates with health-care professionals' attitudes towards the homeless and their interest in working with the homeless population. Moreover, individuals who had more extensive experience with the homeless showed more positive attitudes toward and interest in homeless patients. Therefore, increasing the opportunities to provide direct patient care to the homeless might improve the attitudes of health-care professionals toward this group.

The factor analysis suggested that we do have potential subscales in the instrument that may offer meaningful information regarding the general attitudes of health-care professionals who work with the homeless. The three subscales appear to represent: *personal advocacy*, which contains the items that reflect a personal commitment to work with the homeless; *social advocacy*, which consists of the items that reflect society's responsibility to care for the homeless population; and *cynicism*, which encompasses the items that reflect a negative attitude and a sense of futility in working with the homeless.

A limitation of this study is that only 160 health-care professionals participated in the online administration of the HPATHI. This small sample size limits the effectiveness of the factor analysis. Moreover, our sample focused only on the medical profession as represented by medical students and primary care residents and physicians. The participation of a larger number of health-care professionals from other medical specialties would strengthen the study.

## Conclusions

The development of the HPATHI has many implications for and applications to future research on homeless health care, although its most valuable use may be to assess the attitudes toward homeless persons of medical students, residents, and practicing physicians. Throughout its various iterations, the instrument demonstrated strong internal consistency reliability for the total scale and satisfactory test-retest reliability. The scales identified by the factor analysis also showed satisfactory internal consistency reliability. The information collected from the expert panel and the literature search on attitudes toward the homeless and their health-care status proved to be an excellent framework for determining which statements to retain for the final instrument. The inter-item correlations and the correlations among the subscales indicate that the items are measuring similar underlying constructs within an overall theme – the attitudes of health-care professionals toward the homeless.

Despite the limited sampling, the research process has demonstrated that the HPATHI is a reliable and valid instrument that has the ability to assess the attitudes of health-care professionals toward the homeless population. We believe that the instrument may also be used in the future within the academic framework of medical schools to determine if attitudinal changes are affected by training experiences occurring with the homeless. To determine whether this is the case, we intend to administer the HPATHI as a pre/post-test survey to students who enroll in the homeless health-care track. Moreover, over the next year, we plan to keep on assessing the attitudes of health-care professionals toward the homeless by having new groups respond simultaneously to the ATHI and the HPATHI, as was done in Phase 3 of the original study. Additionally, we intend to include participants from other medical schools in the United States and to expand our sample to other health-care professionals who traditionally work with the homeless, to further test the instrument's overall validity.

## Competing interests

The author(s) declare that they have no competing interests.

## Authors' contributions

DB contributed to the conception, design, and acquisition of data, and drafted and revised the article. FMM contributed to the conception, design, and acquisition of data, helped draft the article, and carried out the data analysis. SK contributed to the conception, design, and acquisition of data, helped draft the article, and carried out the data analysis. DR contributed to the draft of the manuscript and critically revised it for important content. DLC contributed to the conception, design, and acquisition of data. AM contributed to the conception, design, and acquisition of data. RJV contributed to the interpretation of the data and critically revised it for important content. All authors read and approved the final manuscript

## Pre-publication history

The pre-publication history for this paper can be accessed here:



## References

[B1] The Secretary's Work Group on Ending Chronic Homelessness (2003). Ending chronic homelessness: strategies for action.

[B2] Gelberg L, Arangua L, Andersen RA, Rice TH, Kominski GF (2001). Homeless persons. Changing the US Health Care System: Key Issues in Health Services, Policy, and Management.

[B3] Gelberg L, Andersen RM, Leake BD (2000). The behavioral model for vulnerable populations: application to medical care use and outcomes for homeless people [see comments]. Health Serv Res.

[B4] Levy BD, O'Connell JJ (2004). Health Care for Homeless Persons. N Engl J Med.

[B5] Buckner JC, Bassuk EL (1997). Mental disorders and service utilization among youths from homeless and low-income housed families. J Am Acad Child Adolesc Psychiatry.

[B6] Wenzel SL, Koegel P, Gelberg L (1996). Access to substance abuse treatment for homeless women of reproductive age. J Psychoactive Drugs.

[B7] Cheung AM, Hwang SW (2004). Risk of death among homeless women: a cohort study and review of the literature. CMAJ.

[B8] Hwang SW, O'Connell JJ, Lebow JM, Bierer MF, Orav EJ, Brennan TA (2001). Health care utilization among homeless adults prior to death. J Health Care Poor Underserved.

[B9] Elvy A, Brickner PW, Sharer LK, Conanan B, Elvy A, Savarase M (1985). Access to Care. Health Care of Homeless People.

[B10] Hansen JT (2000). Psychoanalysis and humanism: a review and critical examination of integrationist efforts with some proposed resolutions. J Couns Dev.

[B11] Tyson K, Carroll E (2001). Innovative therapeutic care for homeless, mentally ill clients: intrapsychic humanism in a residential setting. Fam Soc.

[B12] Henry-Tillman R, Deloney LA, Savidge M, Graham CJ, Klimberg VS (2002). The medical student as patient navigator as an approach to teaching empathy. Am J Surg.

[B13] Misch DA (2002). Evaluating physicians' professionalism and humanism: the case for humanism "connoisseurs". Acad Med.

[B14] Oreopoulos DG (2001). Compassion and mercy in the practice of medicine. Perit Dial Int.

[B15] Rogers CR (1951). Client-centered Therapy.

[B16] Branch WT, Kern D, Haidet P, Weissmann P, Gracey CF, Mitchell G, Inui T (2001). The patient-physician relationship. Teaching the human dimensions of care in clinical settings. JAMA.

[B17] Kingree JB, Daves WF (1997). Preliminary validation of the attitudes toward homelessness inventory. J Comm Psychol.

[B18] Lester HE, Pattison HM (2000). Development and validation of the Attitudes Towards the Homeless Questionnaire. Med Educ.

[B19] Buchanan D, Rohr L, Kehoe L, Glick SB, Jain S (2004). Changing attitudes toward homeless people. J Gen Intern Med.

[B20] Pill J (1971). The Delphi Method: substance, context, a critique and an annotated bibliography. Socio-Econ Plan Sci.

[B21] Barhhart AJ, Marcy ML, Colliver JA, Verhulst SJ (1995). A comparison of second- and fourth-year medical students on a standardized-patient examination of clinical competence: a construct validity study. Teach Learn Med.

[B22] Last JM, editor (1995). A Dictionary of Epidemiology.

[B23] Clark DL, Melillo A, Wallace D, Pierrel S, Buck DS (2003). A multidisciplinary, leaner-centered, student-run clinic for the homeless. Fam Med.

